# Adverse Effects of High Concentrations of Fluoride on Characteristics of the Ovary and Mature Oocyte of Mouse

**DOI:** 10.1371/journal.pone.0129594

**Published:** 2015-06-08

**Authors:** Songna Yin, Chao Song, Haibo Wu, Xin Chen, Yong Zhang

**Affiliations:** 1 College of Veterinary Medicine, Northwest A&F University, Yangling, 712100, Shaanxi, China; 2 Key Laboratory of Animal Biotechnology, Ministry of Agriculture, Northwest A&F University, Yangling, 712100, Shaanxi, China; Nanjing Agricultural University, CHINA

## Abstract

Reproductive toxicity has been an exciting topic of research in reproductive biology in recent years. Soluble fluoride salts are toxic at high concentrations; their reproductive toxicity was assessed in this study by administering different fluoride salt concentrations to mice. Continuous feeding for five weeks resulted in damage to the histological architecture of ovaries. The expression of genes, including *Dazl*, *Stra8*, *Nobox*, *Sohlh1*, and *ZP3* gene, associated with oocyte formation were much lower in the experimental group as compared with the control group. The number of in vitro fertilization of mature oocytes were also much lower in the experimental group as compared with control. Moreover, the fertility of female mice, as assessed by mating with normal male mice, was also lower in experimental compared with control groups. The expression of the oocyte-specific genes: *Bmp15*, *Gdf9*, *H1oo*, and *ZP2*, which are involved in oocyte growth and the induction of the acrosome reaction, decreased with the fluoride administration. DNA methylation and histone acetylation (H3K18ac and H3K9ac) are indispensable for germline development and genomic imprinting in mammals, and fluoride administration resulted in reduced levels of H3K9ac and H3K18ac in the experimental group as compared with the control group, as detected by immunostaining. Our results indicate that the administration of high concentrations of fluoride to female mice significantly reduced the number of mature oocytes and hampered their development and fertilization. Thus, this study lays a foundation for future studies on fluoride-induced reproductive disorders in women.

## Introduction

Fluoride is an ionic compound of fluorine and occurs naturally at varying levels in rocks, water, and soil because of its high reactivity [1, 2]. A low dosage of fluoride has been established to be beneficial for dental health [3]; however, chronic exposure to large quantities of fluoride interferes with bone formation [4, 5]. Several studies [6, 7] have shown that the anthropogenic introduction of large quantities of fluoride into the environment, for instance, due to the wide usage of fluoride in industrial processes, has adverse effects on plants, animals, and human beings. Fluoride strongly inhibits photosynthesis, lowers IQ, and causes damage to bones, brain, kidney, and spinal cord. Moreover, a review highlights the reduction in fertility caused by fluoride compounds in a majority of the animals species investigated [8]. For investigating the effects of fluoride on humans, mouse is the mainstay of in vitro experimentation as they mirror several aspects of human biology remarkably well. Genome sequencing projects on both humans and mice further support the conservation of biological functions between these two organisms [9, 10]. Several other studies on reproductive disorders in women have extensively employed mouse models [11–13], and the use of mouse models will impact our understanding of ovarian function and fertility in women [11]. Therefore, mouse models have also been employed in this study. The ovary is a functional organ of the female reproductive system and releases a mature oocyte for fertilization. Numerous studies have indicated that exposure to high concentrations of fluoride results in ovarian damage in mice [14, 15]. However, few studies to date explicitly address the exact changes in the expression of several genes. Since epigenetic modifications including genome-wide DNA methylation and histone acetylation have been established to be crucial for normal mammalian development, cellular reprogramming, germline development, and genomic imprinting throughout the mammalian gametogenesis [16], we hypothesized that exposure to fluoride compounds affects in vivo fertilization and in vitro fertilization, and is genetically transmitted to gamete like histone acetylation of the mature oocyte.

## Materials and Methods

### Ethics Statement

All procedures and experiments were approved by the Animal Care Commission of the College of Veterinary Medicine, Northwest A&F University and are in accordance with the Guide for the Care and Use of Laboratory Animals by National Institutes of Health. Healthy mice of the KM strain were purchased from the Experimental Animal Center of The Xi’an Jiaotong University (Xi’an, China) and maintained on a 14/10 h light/dark cycle with free access to food and water in the Laboratory Animal Facility of the College of Veterinary Medicine, Northwest A&F University.

### Animal treatment

One hundred and sixty healthy female (aged 4–6 weeks) and 50 healthy male (aged~ 7~8 weeks) mice of the KM strain were used in this study to estimate the effect of high concentrations of fluoride on ovaries and mature oocytes. The female mice were divided randomly and equally into five groups, as follows: group A(control group) was adminstratered water without fluoride, while group B-E(experimental groups) were administrated 50, 100, 150, 200mg/L NaF in water, respectively. The dosage of NaF was determined on the basis of toxicity of NaF (dissolved in deiondized water) in mice [17, 18]. The administration was continued for 5 weeks.

### Antibodies and reagents

Unless otherwise indicated, reagents were purchased from Sigma Chemical Co. (St. Louis, MO, USA). Mouse monoclonal anti-5-methylcytosine antibody was obtained from Zymo Research Zymo Research Co., (Los Angeles, CA, USA), while rabbit polyclonal anti-H3K9ac, anti-H3K18ac antibodies were purchased from Abcam (Abcam, Cambridge, UK). The secondary antibodies for immunofluorescence, Alexa fluor cy3-labeled goat anti-Rabbit IgG and alexa fluor cy3-labeled goat anti-mouse IgG were purchased from Beyotime Co. (Beyotime, Shanghai, China).

### Collection of mature oocytes and in vitro fertilization

Mature oocytes were obtained from female mice superovulated with 10 IU pregnant mare serum gonadotropin and 10 IU human chorionic gonadotropin, mature oocytes were released from the ampullae of oviducts with H-KSOM at 14~16h after human chorionic gonadotropin administration and cumulus cells were removed by treatment with 1 mg/ml hyaluronidase and pipetting in H-KSOM; Spermatozoa were obtained from the vas deferens of male mice following their sacrifice. In vitro fertilization was carried out by co-incubation of oocytes (numbering ~10–45 from the experimental and control groups) with sperm (2 × 10^5^/mL) in KSOM medium for 4 h [19, 20].

### Estimation of fertility of female mice through in vivo fertilization following mating

Female mice were superovulated with the administration of 10 IU pregnant mare serum gonadotropin and 10 IU human chorionic gonadotropin and subsequently mated with normal healthy male mice. After 20 h, zygotes were released from oviduct ampullae with H-KSOM

### Observation of ovarian tissue by transmission electron microscopy

The ovaries of female mice were removed and fixed with 0.25% glutaraldehyde for 3 h, followed by fixation with 1% osmium tetroxide at 4°C for 2 h. The tissue was washed three times with PBS, dehydrated in 95% and then 100% ethanol, and embedded in Epon 812. Ultrathin sections were stained with uranyl acetate and lead citrate and examined using transmission electron microscope (JEM-2000EX; JEOL, Japan).

### Total RNA extraction and real-time PCR (qPCR)

Total RNA was isolated from each whole ovary using TRIzol reagent (Invitrogen, CA, USA), per the manufacturer’s instructions. First strand cDNA was generated from total RNA using SYBR PrimeScript RT-PCR Kit (Takara BIO Inc., Japan). Mature oocytes were lysed and first-strand cDNA was synthesized directly using SuperScript III CellsDirect cDNA synthesis kits (Invitrogen, CA, USA) according to the report. qPCR was carried out using an ABI StepOnePlus PCR system (Applied Biosystems, CA, USA) under the following conditions: 95°C for 30 s; followed by 40 cycles of 95°C for 5 s, and 60°C for 30 s. SYBR Premix ExTaq II (Takara BIO Inc.) and the Ct method were used to calculate the relative quantity of the target mRNA. Results were normalized to GAPDH and relative to the calibrator, and expressed as fold-change (2^-ΔΔCt^)[21]. All the experiments were repeated three times. The primers we used for qPCR are listed in S1 Table.

### Immunofluorescence Staining

Mature oocytes were fixed with 4% buffered paraformaldehyde solution for 15 min, treated with 0.25% Triton X-100 for 20 min, and blocked with Immunol Staining blocking solution for 2 h; all three steps were carried out at room temperature (RT). The mature oocytes were incubated with the primary antibodies (mouse monoclonal anti-5-methylcytosine antibody, and rabbit polyclonal anti-H3K9ac or anti-H3K9ac antibodies) overnight at 4°C. Following a wash with PBS, the mature oocytes were incubated with the secondary antibodies (Alexa Fluor 488-conjugated goat anti-mouse IgG or goat anti-rabbit IgG) for 2 h at RT and then counterstained with DAPI (blue)/PI (red) to visualise DNA. The mature oocytes were observed and photographed with the aid of a Nikon confocal microscope. The experiment was replicated three times; and four to six oocytes per group were processed in each replication.

### Statistical analysis

All the results are presented as the mean±SD. Data were analyzed by one-way ANOVA and LSD tests using the SPSS13.0 software (SPSS Inc, Chicago, IL, USA). The difference was considered statistically significant at P<0.05.

## Results

### Effect of fluoride administration on ultrastructural features of ovary

**Fig 1:**
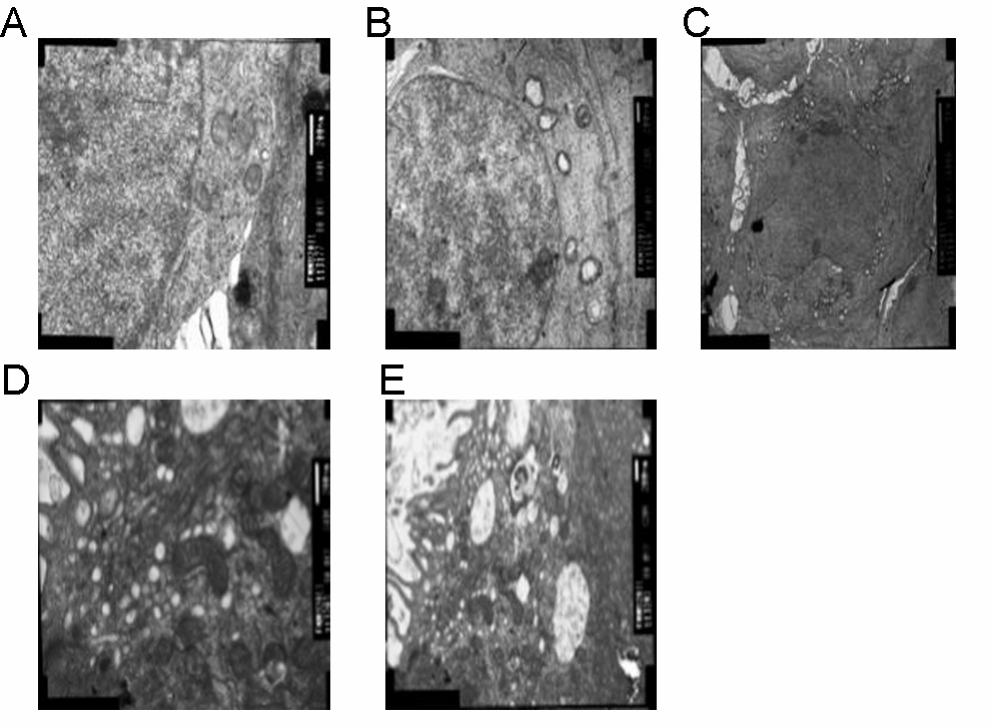
Effect of fluoride administration on ultrastructural features of ovary. (A-E): Ovaries were removed from female mice and ultrathin sections were cut. The histological architecture of ovaries from the control group (A, administered 0 mg/L NaF) and experimental (B-E; administered 50, 100, 150, and 200 mg/L NaF, respectively) groups was examined by transmission electron microscopy.

The effect of fluoride administration on the histological architecture of ovarian tissue was evaluated by observing its ultrastructural features through transmission electron microscopy. The mice in the control group (administered 0 mg/L NaF) exhibited normal ultrastructural features (Fig 1A), while those in the experimental groups with different fluoride concentrations (50, 100, 150, or 200 mg/L NaF), exhibited cellular damage, mitochondrial vacoulation (Fig 1B), or both cytoplasmic and mitochondrial vacoulation (Fig 1C) as a consequence of fluoride exposure. Exposure to higher fluoride concentrations exacerbated cellular damage (Fig 1D and 1E).

### Effect of fluoride administration on expression of germline-specific genes in the ovary

**Fig 2:**
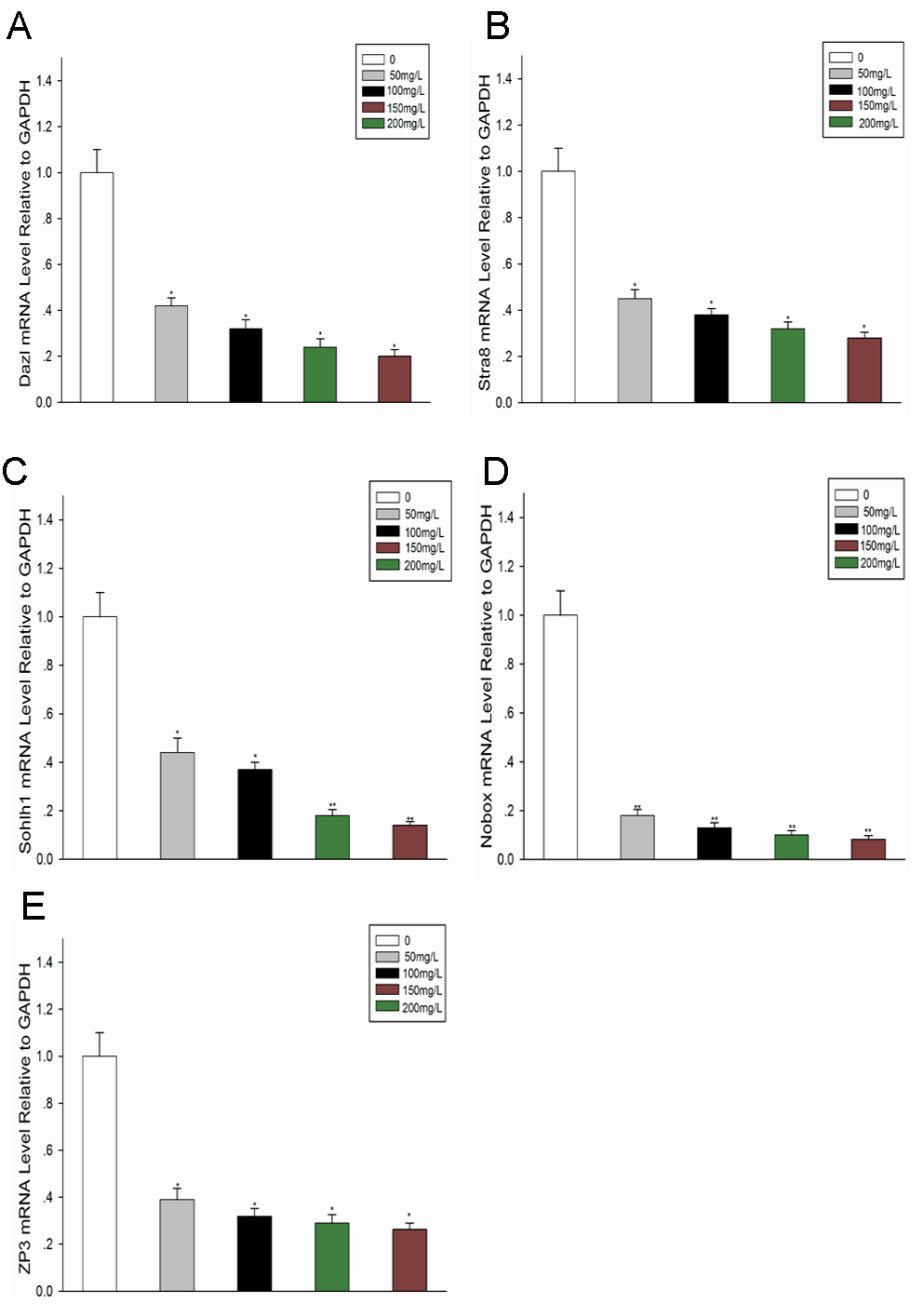
Effect of fluoride administration on expression of germline-specific genes in the ovary. (A-E): mRNA was harvested from ovaries of mice from the control and experimental groups. qPCR was performed for assessing the relative expression levels of germline-specific genes (A: *Dazl*, B: *Stra8*, C: *Sohlh1*, D: *Nobox*, and E: *Zp3*) in the ovary. All data are presented as the mean ± SD and are derived from three independent experiments. *P<0.05; **P<0.01.

RNA was isolated from ovaries of mice from the control and experimental groups, and the expression of potential germline-specific genes, particularly *Dazl*, *Stra8*, *Sohlh1*, *Nobox*, and *Zp3*, was analysed by RT-PCR. As observed in Fig 2A–2E, the expression of these genes was lower in the experimental groups (administered 50, 100, 150, or 200mg/L NaF) compared with the control group (administered 0mg/L NaF). Increase in fluoride concentration resulted in the decreased expressions of these genes, particularly *Nobox*, which was rarely detected in the experimental groups (Fig 2D).

### Effect of fluoride administration on the formation and in vitro/in vivo fertilization of mature oocytes

**Fig 3:**
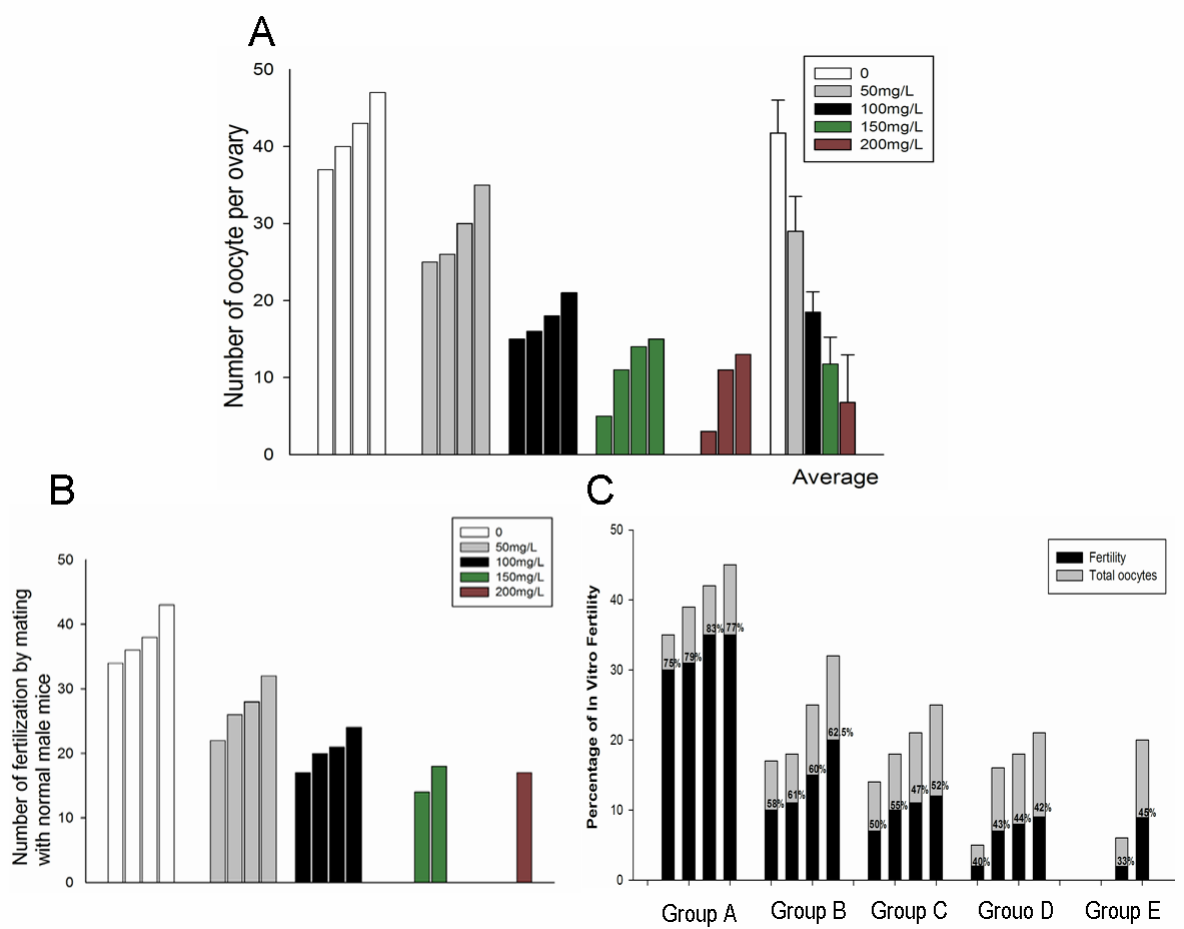
Effect of fluoride administration on formation and in vitro/in vivo fertilization of mature oocytes. Mature oocytes were released from oviduct ampullae of superovulated mice ~14–16 h following the administration of human chorionic gonadotropin, and the number of the mature oocytes in the ovaries (A) and the efficiency of in vitro fertilization (C)were estimated. Mice from the control and experimental groups were mated with normal male mice following the administration of human chorionic gonadotropin for detecting the in vivo fertilization efficiency (B). (Data are presented as mean ± SD, with four mice (*n* = 4) per group).

The effect of high concentrations of fluoride on the formation and in vitro fertilization of mature oocytes was investigated; furthermore, the fertility of female mice exposed to fluorides was examined by mating with normal male mice. Superovulation was achieved by the administration of 10 IU pregnant mare serum gonadotropin and 10 IU human chorionic gonadotropin before mating or harvesting of mature oocytes from the oviduct ampullae, as detailed in Materials and Methods. Fig 3A shows that the number of mature oocytes per ovary was significantly lower in the experimental groups (administered 50, 100, 150, or 200 mg/L NaF) compared with the control group (administered 0 mg/L NaF). This result is also reflected in the lower fertility of fluoride-administered female mice, as assessed by mating with normal male mice (Fig 3B), and in the lower efficiency of in vitro fertilization for the experimental groups compared with the control group (Fig 3C).

### Effect of fluoride administration on the expression of oocyte-specific genes

**Fig 4:**
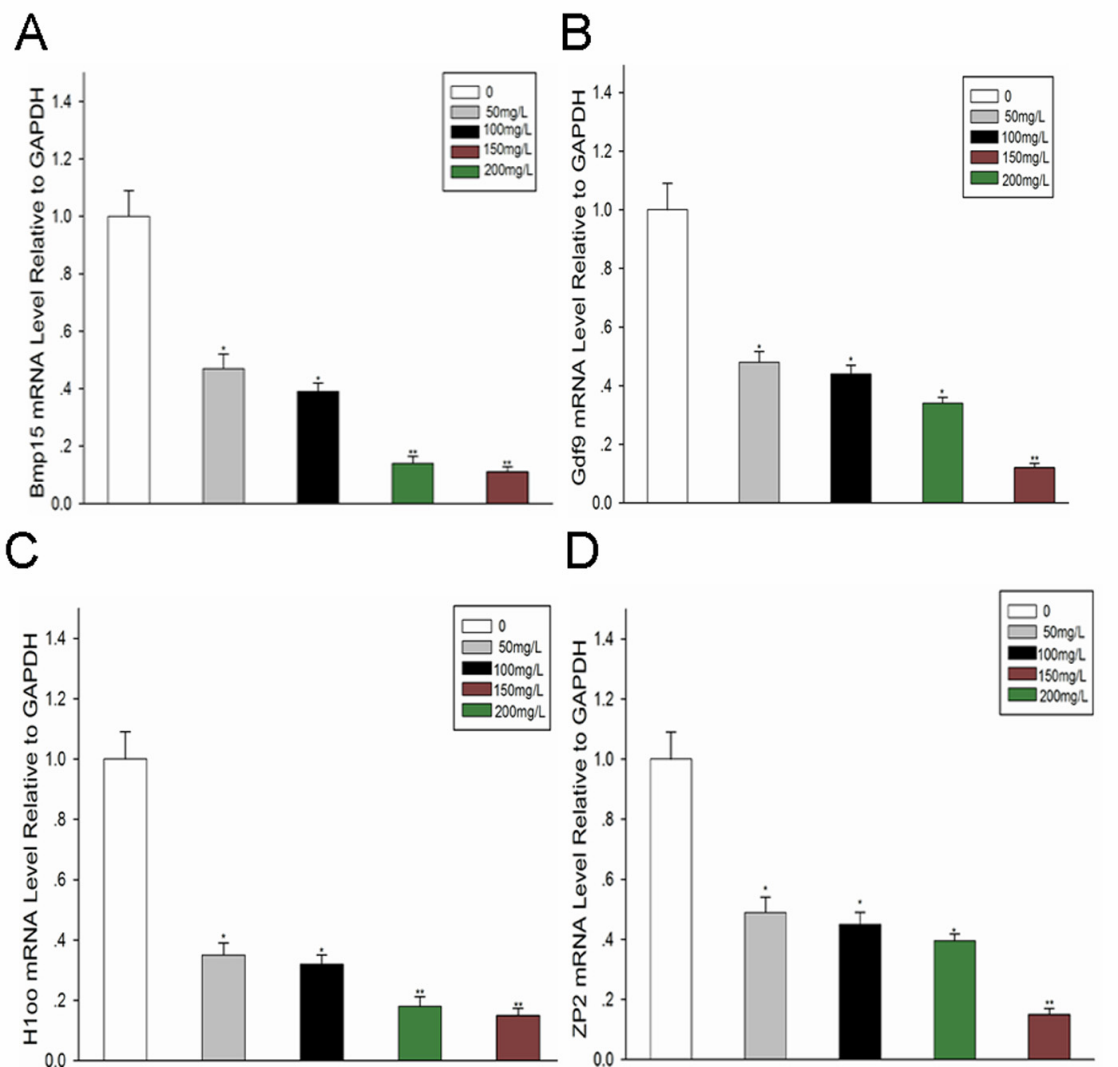
Effect of fluoride administration on the expression of oocyte-specific genes. (A-D): mRNA was harvested from oocytes of mice from the control and experimental groups. RT-PCR was performed for assessing the relative expression levels of oocyte-specific genes (A: *Bmp15*, B: *Gdf9*, C: *H1oo*, and D: *Zp2*) in the oocytes. All data are presented as the mean ± SD and are derived from three independent experiments. *P<0.05; **P<0.01.

The results mentioned above indicate that the number and fertilization of mature oocytes are affected by high concentrations of fluoride. Therefore, the expression of oocyte-specific genes was evaluated by RT-PCR following the direct synthesis of cDNA from mature oocytes, as detailed in Materials and Methods. Four oocyte-specific genes, *Bmp15*, *Gdf-9*, *Zp2*, and *H1oo*, were focused on in this study because of their crucial functions. Expression of all these genes was found to be lower in the experimental groups compared with the control group, with negative association observed between the expression of these genes and fluoride concentration (Fig 4A–4D).

### Effect of fluoride administration on DNA methylation and histone acetylation in mature oocytes

**Fig 5:**
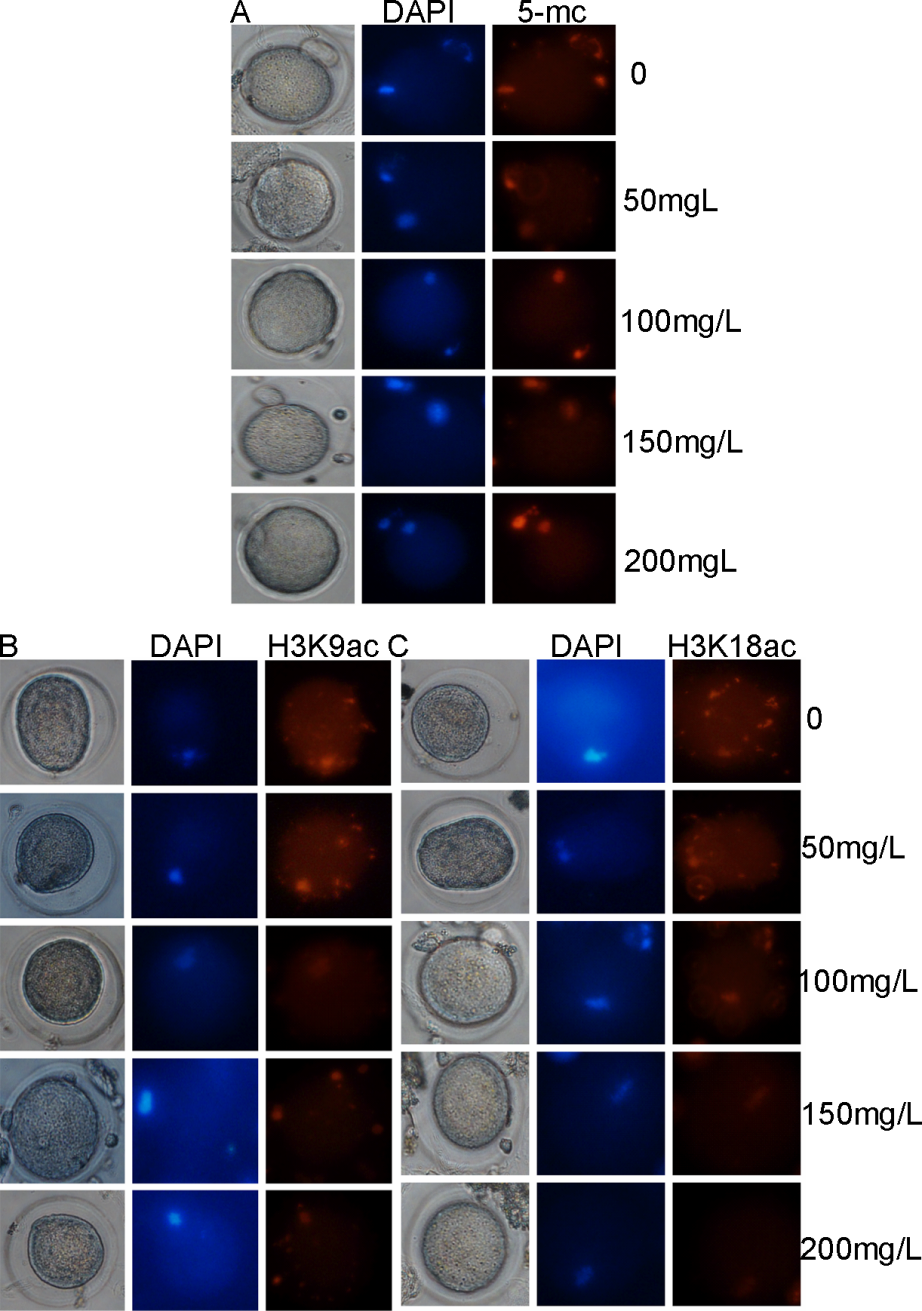
Effect of fluoride administration on DNA methylation and histone acetylation in mature oocytes. Mature oocytes were released from the oviduct ampullae of superovulated mice ~14–16 h following the administration of human chorionic gonadotropin. Immunofluorescence was performed for the detection of levels of 5-methylcytosine (A), H3K9ac (B), and H3K18ac (C). Each sample was stained with anti-5-methylcytosine (green), anti-H3K9ac (green), or anti- H3K18ac (green) antibodies and counterstained with DAPI (blue) to allow DNA visualization. Samples were visualized at (original magnification × 200) for exposure time of 200 ms (anti-5-methylcytosine, anti-H3K9ac and anti-H3K18ac).

Immunostaining was performed to assess the effect of fluoride administration on global DNA methylation and histone acetylation (notably, H3K18ac and H3K9ac) in mature oocytes. As seen in Fig 5A, significant differences were not observed in 5-methylcytosine levels between the experimental (administered various fluoride concentrations) and control groups. In contrast, lower levels of H3K18ac and of the H3K9ac were observed in the experimental groups (Fig 5B and 5C).

## Discussion

Fluorides are well recognized as pollutants, with a great deal of research focused on the environmental hazard that they cause [22, 23]. While the effects of fluoride exposure on fertility are known, its exact effect on the production of mature oocytes in mammalian ovaries remains to be investigated. The objective of this study is to explicitly assess the adverse effects of high concentrations of fluoride on the characteristics of mouse ovary and mature oocyte.

The consumption of large quantities of fluoride administration resulted in obvious damage to the histological architecture of mouse ovaries, as reported previously [14, 24]. Further, the effect of fluoride administration on the expression of germline-specific genes was investigated. Previous studies have reported the association between expression of particular ovary-specific genes and oocyte formation. *Dazl*, expressed during embryonic development in the female gonads of mice well before the onset of meiosis, functions in the first phase of gametogenesis during the differentiation, proliferation and maintenance of primordial germ cells and their substitutes [25]; *Stra8* is required for meiotic progression in the mouse ovary, previous studies demonstrated that meiosis is a sex-specific event where germ cells undergo cellular differentiation to form oocytes or spermatozoa, with abnormal gene expression during meiosis leading to aberrant gamete formation, which is often a major cause of infertility in both males and females [26]; *Nobox* deficiency has been shown to disrupt early folliculogenesis and expression of oocyte-specific genes [27]; *Sohlh1* is a transcription factors of the bHLH family and is specifically expressed in germ cells; it plays a role in oocyte differention, in female, such that *Sohlh1* ablation causes oocyte loss in the neonatal ovary [28]; *Zp3* plays an important role in the development of mouse zona pellucida, which is critical for fertilization [29]. This study revealed that the expression of these genes was much lower in the experimental groups compare with the control group and showed an inverse association with the concentration of fluoride adminstratered. The changes in histological architecture and expression of germline-specific genes in the ovary are likely to affect the formation and fertilization of mature oocytes. The effect of high concentrations of fluoride on the formation of mature oocytes was investigated by inducing superovulation followed by collection of mature oocytes; moreover, in vitro fertilization and in vivo fertilization following mating with normal male mice were also assessed. The results obtained showed that increase in fluoride concentration resulted in lower yield of mature oocytes as well as lower efficiency of in vivo and in vitro fertilization in the experimental groups compared with the control group, which is in agreement with the observed expression of germline-specific genes, as detailed above.

The expression of the following oocyte-specific genes was also assessed following fluoride administration: *Bmp15*, which is involved in oocyte maturation and follicular development; *Gdf-9*, which regulates the oocyte growth and function of oocytes as well as growth and differention of granulose cell; *zp2*, which mediates species-specific sperm binding, induces acrosome reaction, and prevents post fertilization polyspermy; and *H1oo*, whose expression is restricted to the growing/maturing oocyte and to the zygote [30]. The expression of these oocyte-specific genes was decreased upon fluoride administration, which is expected to disrupt the normal maturation of oocyte.

The important role played by histone acetylation and DNA methylation in oogenesis is widely accepted. Previous studies have shown that occurrence of 5-methylcytosine in mammals genomes is crucial for normal mammalian development, while histone acetylation is associated with a transcriptionally active state and allows access of transfactor to DNA sequence. Abnormal epigenetic modification is expected to be detrimental to offspring as a consequence of DNA damage [31]. Therefore, the levels of global DNA methylation, and the active histone marks H3K9ac and H3K18ac were assessed in mature oocytes following the administration of fluoride to mice. The results revealed the absence of significant differences in the level of 5-methylcytosine between the experimental and control groups. However, the levels of H3K9ac and H3K18ac were lower in the experimental compared with the control groups and decreased with increase in fluoride concentration. Such abnormal epigenetic modification is likely to be particularly detrimental to offspring.

Behavioral differences were also observed in mice belonging to various experimental groups. Mice belonging to the experimental group D (administered 150 mg/L NaF) were observed to be thinner compared with the other groups, while the mice of group E (administered 200 mg/L NaF) consumed a much greater quantity of water; moreover, the mice of groups C, D, and E (administered 100, 150, and 200 mg/L NaF, respectively) displayed a tendency to closely approach one another. This is attributable to the neurotoxicity and behavioral changes caused upon fluoride consumption in animals [32, 33].

Taken together, this study suggests that the administration of high concentrations of fluoride to female mice not only results in ovarian damage but also significantly reduces the number and the fertilization potential of mature oocytes by reducing the expression of genes that play an important role in the normal development and maturation of oocytes. The results obtained in this study could thus be employed for statistical analysis of the association between exposure to high concentrations of fluoride and reproductive disorders in women.

## Supporting Information

S1 Table The sequences of primers used for SYBER Green real-Time PCR. The special primers for *GAPDH*, *Dazl*, *Stra8*, *Sohlh1*, *Nobox*, *Zp3*, *Bmp15*, *Gdf9*, *H1oo*, and *Zp2* were designed respectively according to the reported sequences.
(DOCX)
