# Supplementary material for: Adverse Effects of High Concentrations of Fluoride on Characteristics of the Ovary and Mature Oocyte of Mouse
Source: PLoS One. 2015 Jun 8;10(6):e0129594. doi: 10.1371/journal.pone.0129594 (PMC4460091; doi:10.1371/journal.pone.0129594)
Supplement: S1 Table — The special primers for GAPDH, Dazl, Stra8, Sohlh1, Nobox, Zp3, Bmp15, Gdf9, H1oo, and Zp2 were designed respectively according to the reported sequences. (DOCX) [file pone.0129594.s001.docx]

**Table S1 Sequences of primers for Real-Time PCR**

| **Gene Prime name Sequence(5’—3’) Product length**  **(Accession number)** |
| --- |
| ***GAPDH* F GTGTTCCTACCCCCAATGTGT 248 bp**  **(NM_008084) R ATTGTCATACCAGGAAATGAGCTT**  ***ZP2*  F GTGGCAGAGGAAAGCATCTGT 111bp**  **(**[**NM_011775**](http://www.ncbi.nlm.nih.gov/entrez/query.fcgi?cmd=Search&db=Nucleotide&term=NM_011775)**) R GACTGAGGAAGGCTTACTGAGT**  ***Gdf-9* F TCTTAGTAGCCTTAGCTCTCAGG 116bp**  **(**[**NM_008110**](http://www.ncbi.nlm.nih.gov/entrez/query.fcgi?cmd=Search&db=Nucleotide&term=NM_008110)**) R TGTCAGTCCCATCTACAGGCA**  ***Bmp15***  **F TCCTTGCTGACGACCCTACAT 100bp**  **(**[**NM_009757**](http://www.ncbi.nlm.nih.gov/entrez/query.fcgi?cmd=Search&db=Nucleotide&term=NM_009757)**) R TACCTCAGGGGATAGCCTTGG**  ***H1oo*  F GTTGCCGCAGAATCCAAGC 103bp**  **(**[**NM_**](http://www.ncbi.nlm.nih.gov/entrez/query.fcgi?cmd=Search&db=Nucleotide&term=NM_009757)**138311) R GCTACAACTGATGTGCCCTGG**  ***Dazl*  F ATGTCTGCCACAACTTCTGAG 170bp**  **(**[**NM_**](http://www.ncbi.nlm.nih.gov/entrez/query.fcgi?cmd=Search&db=Nucleotide&term=NM_009757)**010021) R CTGATTTCGGTTTCATCCATCCT**  ***Stra8* F ACAACCTAAGGAAGGCAGTTTAC 173bp**  **(**[**NM_**](http://www.ncbi.nlm.nih.gov/entrez/query.fcgi?cmd=Search&db=Nucleotide&term=NM_009757)**009292) R GACCTCCTCTAAGCTGTTGGG**  ***Nobox* F AAGACCCGAACCCTGTACC 93bp**  **(**[**NM_**](http://www.ncbi.nlm.nih.gov/entrez/query.fcgi?cmd=Search&db=Nucleotide&term=NM_009757)**130869) R CTCATGGCGTTTGTCACTGTC**  ***Sohlh1* F GATGTCTGTGTACTTCCTCC 234bp**  **(**[**NM_001001714**](http://www.ncbi.nlm.nih.gov/entrez/query.fcgi?cmd=Search&db=Nucleotide&term=NM_001001714)**) R CTGGCTCACTGAATGACAAC**  ***ZP3*  F ATGGCGTCAAGCTATTTCCTC 186bp**  **(**[**NM_**](http://www.ncbi.nlm.nih.gov/entrez/query.fcgi?cmd=Search&db=Nucleotide&term=NM_009757)**011776) R CGTGCCAAAAAGGTCTCTACT** |
